# Infratentorial ependymomas—a study of the centre in Katowice

**DOI:** 10.1007/s00381-015-2683-9

**Published:** 2015-03-31

**Authors:** Marek Mandera, Joanna Makarska, Grażyna Sobol, Katarzyna Musioł

**Affiliations:** 1 Department of Emergency Medicine and Paediatric Neurosurgery, Medical University of Silesia, ul. Medyków 16, Katowice, Poland; 2 Department of Oncology, Hematology and Chemotherapy, Medical University of Silesia, ul. Medyków 16, Katowice, Poland

**Keywords:** Infratentorial ependymoma, Children, Treatment, Results

## Abstract

The aim of the study was to assess the correlation of the results of the treatment of infratentorial ependymomas with the degree of resection and histopathological diagnosis. The study was conducted on a group of 19 patients, 13 boys and 6 girls aged 3 months to 16 years, with infratentorial ependymoma treated at the Department of Paediatric Neurosurgery of the Medical University of Silesia in Katowice from January 2000 until December 2008. The most significant factor having an impact on overall survival and progression-free survival was totality of tumour resection. There has been no statistically significant influence of the histopathological type of ependymoma on the result of treatment. The tendency to report better results of treatment of non-anaplastic ependymoma seems to derive from a statistically higher frequency of total removal of tumours of this type.

## Introduction

Ependymoma is a neoplasm of the central nervous system that arises from ependymal cells that line the ventricles of the brain and the centre of the spinal cord. Intramedullary, spinal cord ependymomas occur more frequent in adults than in children, whereas the highest rate of occurrence of intracranial ependymomas has been noted in patients aged less than 20. Along with medulloblastoma and pilocytic astrocytoma, intracranial ependymoma is the most frequent primary tumour of the primary nervous system. It has been estimated that the frequency of occurrence of intracranial ependymoma amounts to 6–10 % of all intracranial tumours [1, 2]. Being located in the fourth brain ventricle, intracranial ependymomas are usually infratentorial and constitute one of the most frequent tumours of the posterior cranial fossa. WHO grading of ependymal tumours includes the following types of ependymomas: ependymomas (cellular ependymoma, papillary ependymoma, clear cell ependymoma, tanycytic ependymoma) graded WHO II, anaplastic ependymomas graded WHO III, and myxopapillary ependymomas and subependymomas graded WHO I. The selection of optimised treatment of ependymomas, especially in the case of treatment of children, involves a lot of controversy and varies from medical centre to medical centre. The treatment involves surgical removal of the tumour, radiation therapy and chemotherapy [1–3].

## The aim of the study

The aim of the study was to assess the correlation of the results of the treatment of infratentorial ependymomas with the degree of resection and histopathological diagnosis.

## Material and methods

The study was conducted on a group of 19 patients with infratentorial ependymoma treated at the Department of Paediatric Neurosurgery, Medical University of Silesia in Katowice from January 2000 to December 2008. Firstly, the medical documentation of 360 patients treated for intracranial tumours was subject to retrospective analysis. Out of these patients, a group of 19 patients with infratentorial ependymoma was selected, including 13 boys and 6 girls aged 3 months to 16 years. Secondly, the medical documentation was analysed with regard to gender, age and types of applied treatment with a special focus on the degree of resection, histopathological diagnosis, progression-free survival (PFS) and overall survival (OS). The degree of tumour resection was assessed on the basis of the MRI examination performed 6 weeks after the surgery. Lack of features of tumour residue was taken as the criterion of total resection of the tumour. Progression-free survival (PFS) was defined as the period from diagnosis to reported progression, recurrence of the disease or death. Overall survival (OS) was defined as the period from diagnosis to death of any cause. In order to assess progression-free survival and overall survival, the Kaplan-Meier method was applied. Log-rank test was applied to analyse progression-free survival and overall survival with regard to histopathological type and degree of tumour resection. The results were statistically analysed by means of a specialist software Statistica PL v 6.0, and the level of significance assumed was *α* ≤ 0.05.

## Results

Among 140 patients with posterior cranial fossa tumours that were treated at the Department of Paediatric Neurosurgery in Katowice, there were 19 (12.8 %) cases of infratentorial ependymoma. Infratentorial ependymoma was diagnosed in 13 (68.4 %) boys and 6 (31.6 %) girls. The relation of young male patients to young female patients was 2.16. The age of the children at the moment of diagnosis varied from 3 months to 16 years. Five children were younger than 4 when their disease was diagnosed. All of the children were subject to surgical treatment. Total resection was achieved in 11 (57.9 %) patients, whereas subtotal resection in 7 (36.8 %) patients. One patient (5.2 %) was subject to tumour biopsy.

Five patients were subject to second surgery due to tumour recurrence: Three children were operated twice, one child was operated three times and also one child was operated five times. Histopathological examination resulted in a diagnosis of anaplastic ependymoma in 11 (57.9 %) patients. Moreover, ependymoma was diagnosed in 8 (42.1 %) patients. In 6 (31.6 %) children older than 4 with diagnosed anaplastic ependymoma and reported tumour residue or tumour progression, radiation therapy was applied. In nine (47 %) children with diagnosed anaplastic ependymoma and reported tumour residue or tumour progression, chemotherapy was applied.

**Fig. 1:**
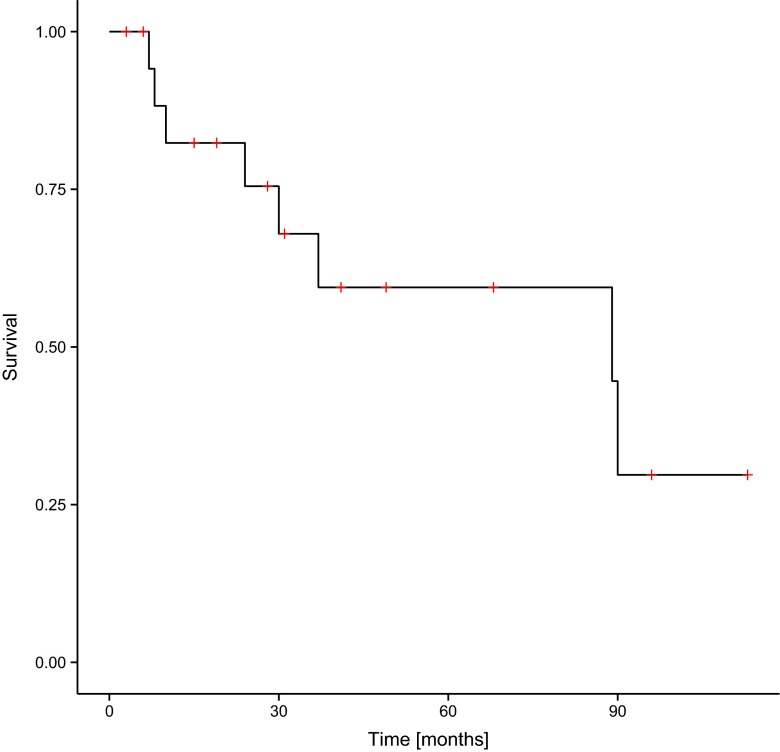
Progression-free survival (PFS) in the group of patients involved in the study

**Fig. 2:**
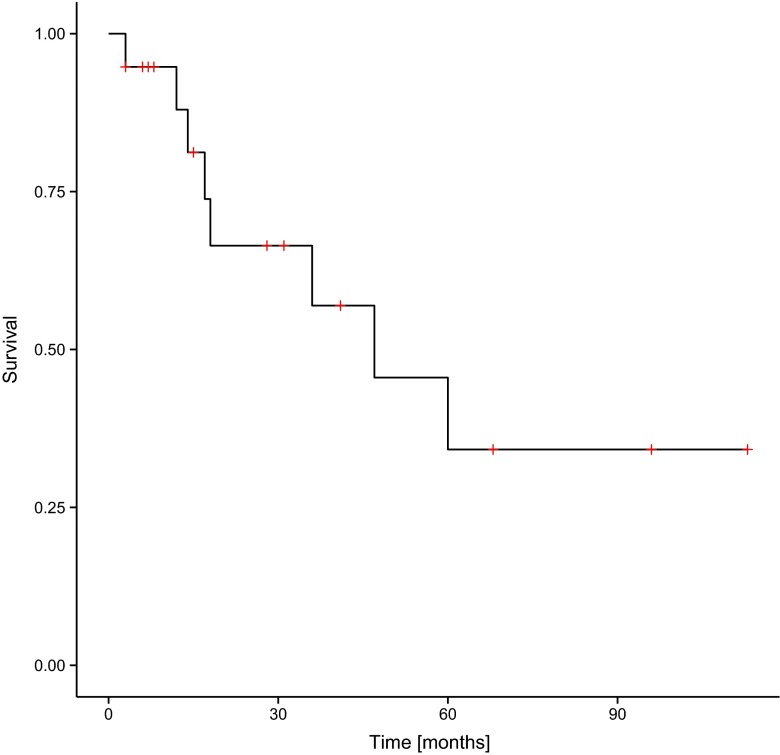
Overall survival (OS) in the group of patients involved in the study

The time of observation varied from 3 to 113 months (40.2 months in average). Tumour progression or tumour recurrence was noted in eight (42.1 %) patients within the period of 3 to 60 months. The average time until progression or recurrence amounted to 25.8 months. There were eight (42.1 %) patients who died after an average time of 37 months (from 6 to 90 months), one of them (12.5 %) with diagnosed ependymoma and seven of them (87.5 %) with diagnosed anaplastic ependymoma. All deaths were pronounced in a group of patients with subtotal resection. Progression-free survival (PFS) and overall survival (OS) were assessed in the group of patients, which are illustrated in Figs. 1 and 2.

**Fig. 3:**
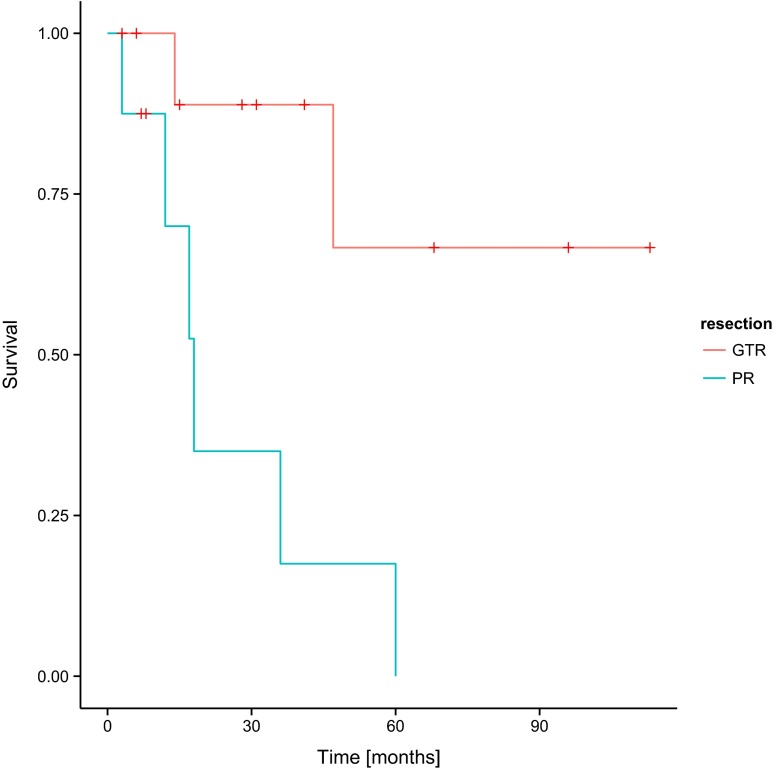
Progression-free survival (PFS) and the degree of tumour resection in the group of patients involved in the study. *GTR* gross tumour resection, *PR* partial resection

**Fig. 4:**
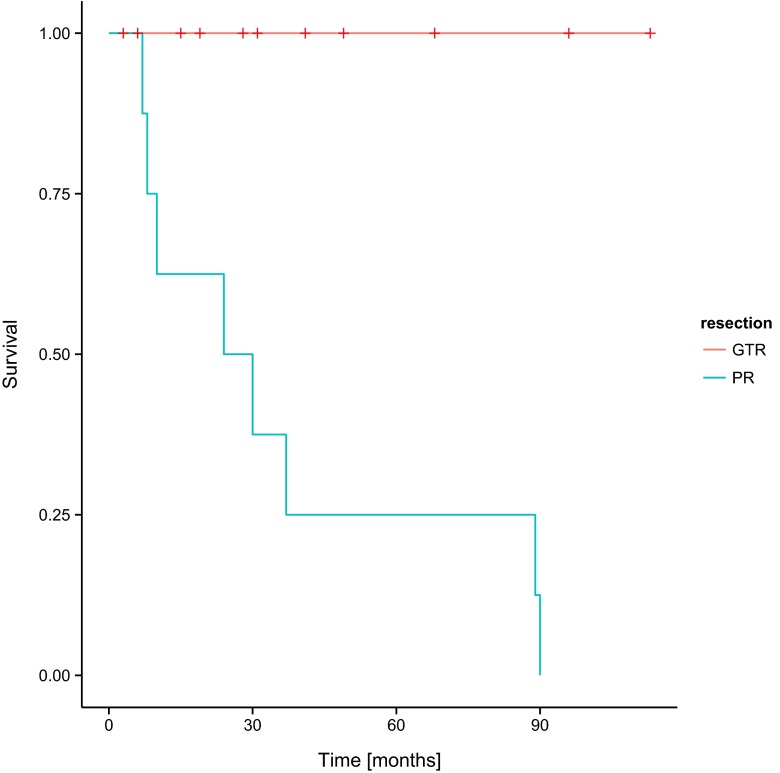
Overall survival (OS) and the degree of tumour resection in the group of patients involved in the study. *GTR* gross tumour resection, *PR* partial resection

It has been reported that the totality of tumour resection is an independent and positive factor having an impact on PFS and OS. This is illustrated in Figs. 3 and 4. The progression of the disease was observed in two children who underwent total tumour resection (respectively 8 and 43 months after surgery). No deaths were pronounced in the group of patients with total resection during observation.

**Fig. 5:**
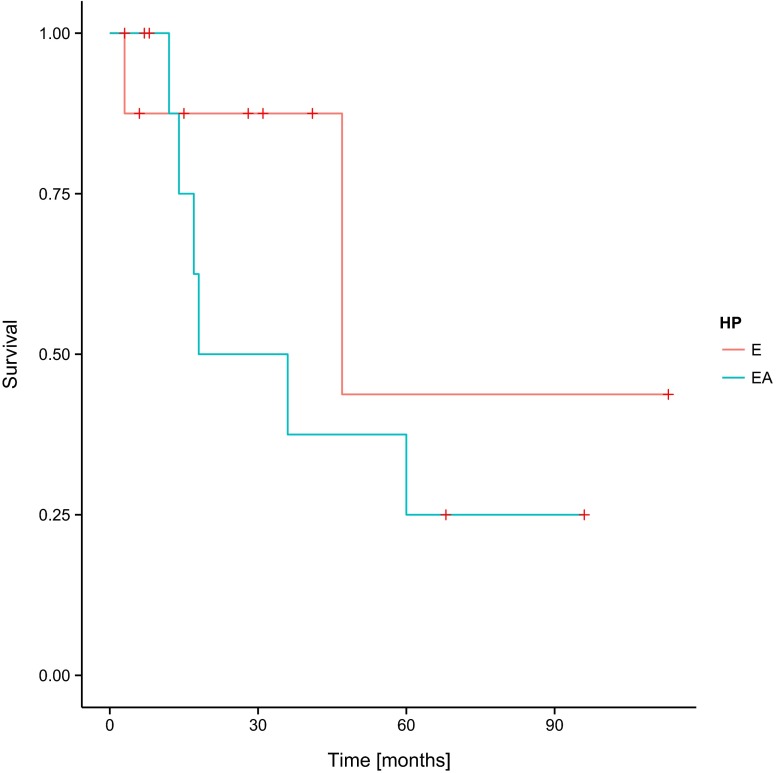
Progression-free survival (PFS) and histopathological diagnosis in the group of patients involved in the study—log-rank test *p* = 0.31704. *E* ependymoma, *AE* anaplastic ependymoma

**Fig. 6:**
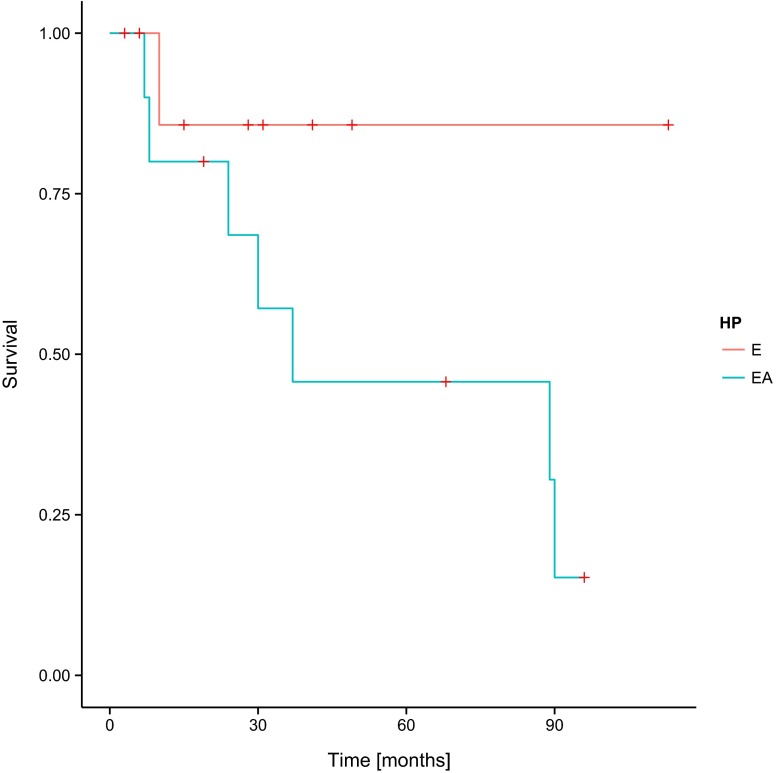
Overall survival (OS) and histopathological diagnosis in the group of patients involved in the study—log-rank test *p* = 0.11713. *E* ependymoma, *AE* anaplastic ependymoma

In the assessment of the correlation of PFS and OS with the histopathological type of tumour, no statistically significant influence of the histopathological type of ependymoma on the results of the treatment has been noted (Figs. 5 and 6).

**Fig. 7:**
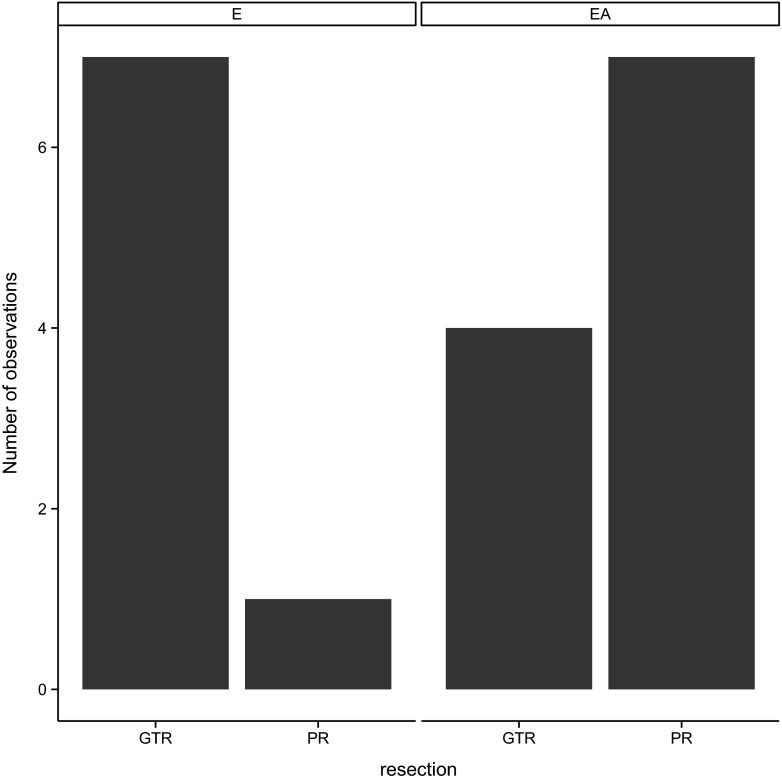
Histopathlogical diagnosis and the degree of tumour resection in the group of patients involved in the study—Pearson’s chi-squared test *p* < 0.005. *GTR* gross tumour resection, *PR* partial resection, *E* ependymoma, *AE* anaplastic ependymoma

It has been noted that the treatment is more effective in the case of ependymoma grade II. Only one patient with ependymoma grade II died, while there were seven deaths in the group of anaplastic ependymoma. On the other hand, however, the total tumour resection was obtained in seven of eight patients with ependymoma, while in the group of anaplastic ependymoma, only in 4 of 11 cases. Better results of the treatment of non-anaplastic ependymoma seem to derive from the fact that such ependymoma was more frequently completely removed (Fig. 7).

## Discussion

Intracranial ependymomas are the most frequent primary tumours of the central nervous system in children. These tumours are characterised by uncertain prognosis, and the types of treatment vary from centre to centre. In our study, infratentorial intracranial ependymoma constituted 13 % of all treated tumours of the posterior cranial fossa. They were most frequent in boys, and the average age of patients when the disease had been diagnosed was 7.7. Surgical treatment is the basic method of treatment of ependymoma. All children treated for ependymoma at the Department of Paediatric Neurosurgery in Katowice were subject to a surgery. In more than half of the children (58 %), total resection was performed, while in almost 37 %, resection was subtotal, and in 5 %, biopsy was selected as treatment. It is generally known that one of the most significant factors influencing prognosis in patients with primary ependymoma is the degree of resection. It is also in our study that the totality of tumour resection proved to be an independent factor that influenced progression-free survival and overall survival in a positive way. Similarly, the observation carried out by Phi et al. [4] revealed significantly longer PFS in the group of children who underwent total resection of the tumour (*P* = 0.029).The analysis of 182 cases of ependymoma treatment available in medical literature by Cage et al. [1] resulted in a similar conclusion. In patients after total tumour resection, the longest period of PFS and OS was noted independently of location of the tumour and its histopathological type. In contrast to this, subtotal tumour resection involved higher mortality rate that was minimised by additional radiation therapy in the case of ependymoma graded WHO III [1]. Similarly in our study, all deaths were pronounced in a group of patients who had been subject to subtotal resection. It should be added that radiation therapy was applied in six (31.6 %) children older than 4 with diagnosed anaplastic ependymoma and reported tumour residue or progression. This procedure was conducted in compliance with the “Standardised and Modified Programme of Diagnostics and Treatment of Tumours of the Central Nervous System in Children” recommended by the Polish Paediatric Solid Tumour Group. The role of chemotherapy has been another broadly discussed issue with regard to the treatment of ependymoma. Chemotherapy has been suggested to decrease tumour mass, increase resectability and to avoid the necessity of application of radiation therapy in infants [4]. In our study based on the guidelines of the “Standardised and Modified Programme of Diagnostics and Treatment of Tumours of the Central Nervous System in Children,” chemotherapy was applied in nine (47 %) children older than 4 with diagnosed anaplastic ependymoma and reported tumour residue or tumour progression. In the treatment of ependymoma, especially recurring ependymoma, stereotactic radiosurgery is additionally applied after resection and conventional radiation therapy. Kano et al. [5] reported that, with regard to ependymoma of small size, the effectiveness of recurrence or progression treatment was increased owing to the application of stereotactic radiosurgery. A more positive response to treatment involving stereotactic radiosurgery was also observed in children with a late disease recurrence [6]. In spite of the development of alternative methods of treatment of ependymoma, such as chemotherapy, conventional radiation therapy and stereotactic radiosurgery, surgical treatment is still the basis of treatment procedures both in primary and recurrent tumour [2]. In the group of 19 patients treated for ependymoma at the Department of Paediatric Neurosurgery in Katowice, tumour recurrence was noted in 40 % (eight) of patients after an average 25.5-month period. Out of this group of eight children, five patients were subject to secondary surgery. Similar results have been reported by Vinchon et al. [2], who diagnosed recurrence in 33 out of 70 patients (47 %) treated for intracranial ependymoma, where 18 patients were subject to another surgery. Taking into account a 5-year period of time, the survival rate in the group of patients after secondary surgery amounted to 38 %, whereas in the case of patients after total resection, the survival rate was as high as 58 %. From the statistical perspective, OS in patients with tumour recurrence was significantly longer in patients after surgical treatment. Moreover, a longer OS was noted in patients who were subject to total resection during the second surgery. That perioperative mortality was lower in the case of second surgery than in the case of the first surgery that seems to be an interesting observation. Another intriguing point is that, in spite of the decrease in body condition caused by scars remaining after the primary surgery and radiation therapy after it, the period of hospital stay was shorter after second surgery than after the first surgery. Such observations may result from a lower tumour mass and/or a better preparation of the patient for the second surgery [2]. It was also in the study by Zacharouilis et al. [7] that better prognosis in the treatment of recurrent ependymomas was noted in a group of patients after gross total resection. A significantly longer time of overall survival was reported in a group of patients subjected to gross total resection than in patients after subtotal resection (44 vs. 23 %). However, no correlation of histopathological type of tumour and the results of treatment were reported with regard to both primary tumour—and recurrent tumour surgery [2, 3, 8]. In our study, the correlation of PFS and overall survival (OS) with histopathologial type of the tumour was analysed. However, no statistically significant influence of the histopathologial type of the tumour on the result of treatment was noted. Similarly, in a retrospective analysis of histopathological tests in 66 children who were subject to surgical treatment of ependymoma, Gerszten et al. [8] did not find any histopathological parameter that might have a prognostic value. What they reported was only that necrosis, nuclear pleomorphism and proliferation of blood vessels in tumour tissue correlate with a shorter PFS. This correlation, however, was not statistically significant [8]. A similar observation was made by Pollack et al. [3], who reported that both dissemination of tumour cells and the presence of anaplastic features, such as high mitotic index, did not have influence on the outcome. Such factors as degree of tumour resection, age of the child and the period of occurrence of symptoms until diagnosis, on the contrary, had a prognostic value. Nevertheless, in our study, better results of treatment of non-anaplastic ependymoma were reported, which may derive from significantly higher frequency of total removal of tumours of this type. What is more, an insufficient or non-representative tumour specimen and/or not standardised criteria of histological assessment applied in different medical centres are additional factors that may independently decrease prognostic value of the histopathological test [9]. To add to this, it needs to be pointed out that some studies reported a correlation of the histopathological character of tumour with treatment [9]. For example, Phi et al. [4], in their multivariate analysis of 33 children with infratentorial ependymoma, showed that the shorter PFS correlated with anaplastic histology of the tumour (*P* = 0.004), a higher mitotic counts (*P* = 0.001) and a higher Ki-67 index (*P* = 0.004). In the course of treatment of ependymomas, genetic aberrations occurring in tumour cells may also function as prognostic factors. Korshunov et al. [9] based their study on gene expression profiles and classified the analysed cases of ependymoma with regard to location of the tumour, progression rate and age of the patient. In their research based on the analysis of the genetic profile, they also identified genes correlating with better prognosis. Homozygous deletion of the gene CDKN2A as well as chromosome 1q duplication constituted the best independent indicators of unfavourable prognosis, whereas duplication of chromosomes 9, 18 and 15q, and chromosome 6 deletion indicated favourable prognosis [9, 10]. Another independent prognostic factor is human telomerase reverse transcriptase (hTERT) expression. Having based their assessment on immunohistochemical tests, Tabori et al. [10] evaluated hTERT expression in a group of 65 patients who were subject to surgery in order to treat ependymoma. A 5-year PFS was noted in 57 % of hTERT-negative patients and 21 % of hTERT-positive patients. As far as 5-year OS is concerned, it was reported in 84 and 41 % of patients respectively. Both confirmed the prognostic value of hTERT expression [11]. Wani et al. [12] evaluated the genetic profile of 56 patients treated for infratentorial ependymoma. Two groups of patients were taken into consideration: short recurrence-free survivors (PFS shorter than 3 years) and long recurrence-free survivors (PFS longer than three years). The aim of the study was to develop a clinical test for the routine assessment of patients diagnosed with ependymoma. The analysis resulted in selecting a 10-gene signature which was an independent predictor of PFS. The authors demonstrated also that a shorter survival time was associated with increased expression of genes associated with angiogenesis and proliferation [13]. Genetic markers added to usually applied clinical and histopathological prognostic factors may result in easier prognosis in the treatment of ependymoma. Unfortunately, an important factor hindering the common use of molecular prognostic tests is their high cost.

## Conclusion

1. The most significant factor having an impact on overall survival and progression-free survival is totality of tumour resection.
2. There has been no statistically significant influence of the histopathological type of ependymoma on the result of treatment.
3. The tendency to report better outcome of non-anaplastic ependymoma seems to derive from a statistically higher frequency of total removal of tumours of this type.
